# Effective Removal of Cadmium Ions from a Simulated Gastrointestinal Fluid by *Lentinus edodes*

**DOI:** 10.3390/ijerph111212486

**Published:** 2014-12-01

**Authors:** Xin Qiao, Wen Huang, Yinbing Bian

**Affiliations:** 1 College of Food Science and Technology, Huazhong Agricultural University, Wuhan 430070, China; E-Mail: qiaoxinspring@163.com; 2 Institute of Applied Mycology, Huazhong Agricultural University, Wuhan 430070, China; E-Mail: bianyb.123@163.com

**Keywords:** adsorption, antidote, cadmium, heavy metal, *L. edodes*, simulated gastrointestinal fluid

## Abstract

*Lentinus edodes*, a functional food, was evaluated as a potential antidote for adsorption/removal of cadmium ion from simulated gastrointestinal fluids. An adsorption/removal capacity of 65.12 mg/g was achieved by *L. edodes* in solutions with a pH ranging from 2.5 to 6.0, while little if any adsorption was observed in solutions with a pH under 2.5. In solutions with pH 6.0, 84% of the cadmium adsorption by *L. edodes* occurred in the first minute. Scanning electronic microscopic examination showed that the cell wall polysaccharides of *L. edodes* provided a rough sponge-like surface for effective cadmium adsorption. FTIR indicated that the carboxyl, hydroxyl and –NH groups of the cell wall polysaccharides and proteins were the primary functional groups that chemically bind with cadmium ions. The energy dispersive spectrometry further revealed that cation exchange might be attributed to cadmium biosorption. These results suggested that *L. edodes* was effective for cadmium detoxication, especially in low concentration.

## 1. Introduction

Cadmium pollution has become a serious issue in recent years [1]. Uptake of this toxic compound can result in multiple human health problems such as lung insufficiency, cardiovascular system disturbances, liver and kidney damage and cancer [2,3,4]. How to prevent and/or minimize its damage in case of uptake is becoming a worldwide research interest. Application of complex chelating agents is the current approach to treat cadmium poisoning. For example, British anti-Lewisite, dimercapto-succinic acid (DMSA), dimercaptopropanesulfonate (DMPS) and ethylenediaminetetraacetic acid (EDTA) are used to treat patients suffering from heavy metal intoxication [5,6,7,8,9]. Upon administration, these chelating complexes bind with heavy metals such as cadmium, that will be removed eventually through the kidney. However, this approach requires multiple administrations which may cause a burden to and damage the renal system during the clearance of metal chelates from the body. In addition, these chelating agents are capable of binding essential metals, resulting in severe mineral deficiency and anemia [8,10,11]. Therefore, it is imperative to develop an effective and safe detoxification agent for the treatment of cadmium intoxication.

*L. edodes* has been recognized as a healthy food. It is rich in nutrients, with a unique flavor, and it contains a variety of physiological active substances. More importantly, its capacity for adsorbing heavy metals is much higher than that of green plants and animal-derived food [12,13]. For the above-mentioned reasons, it was of interest to evaluate *L. edodes* as a potential chelating agent for the therapeutic treatment of cadmium poison without significant side effects. To this end, we have characterized the capacity of the *L. edodes* for adsorption/removal of cadmium from simulated gastrointestinal fluids.

## 2. Materials and Methods

### 2.1. Preparation of L. edodes Powder and Cadmium Solution

*L. edodes* (L26) was collected from the Institute of Applied Mycology, Huazhong Agricultural University, Wuhan, China. The collected samples were washed multiple times using sterile physiological saline, oven-dried at 80 °C for 24 h, and grounded using a roll crusher. The grounded *L. edodes* powder with a certain size were obtained through a plastic sieve, and stored for use as antidote.

A stock solution (1000 mg/L) of Cd(II) was prepared by dissolving CdCl_2_ (analytical grade) in deionized water. The stock solution was diluted as needed for specific experiments.

### 2.2. Effect of pH on Adsorption

A solution containing 100 mg/L cadmium was made from the stock solution, and then divided into seven equal volumes (500 mL). The pH of these seven solutions was adjusted to 2.5, 3, 4, 5, 6, 7 and 8, respectively, using 1 M HCl or NaOH. Each solution was mixed with *L. edodes* powder (<100 μm; 1 g/L), incubated for 2 h (140 rpm, 37 °C). After centrifugation, the supernatant was analyzed for the remaining cadmium.

### 2.3. Effect of Powder Size

Three diluted cadmium solutions (50, 100 and 200 mg/L) were prepared from the stock solution. After pH adjustment (6.0), each diluted solutions were divided into 13 equal volumes (500 mL). Three sets of antidote were weighted; with each set containing 4 samples with different size of *L. edodes* powder (<100, 100–125, 125–150, 150–180 and 180–210 μm; 1 g/L). These antidotes were then added into the corresponding cadmium solution (39 samples). These samples were then incubated for 2 h (140 rpm, 37 °C). After centrifugation, the supernatant was analyzed for the remaining cadmium.

### 2.4. Effect of Initial Cadmium Ion Concentration

A series of solutions were prepared, with each containing 5, 10, 20, 50, 100, 200, 300, 400, 500, 600 mg/L cadmium, respectively. After pH adjustment (6.0), these solutions were mixed with the *L. edodes* antidote (<100 μm; 1 g/L). The mixture was incubated for 2 h (140 rpm, 37 °C). These samples were then centrifuged; and the supernatant was analyzed for the remaining cadmium.

### 2.5. Cadmium Adsorption Time Course

Three cadmium solutions (50, 100 and 200 mg/L) was made from the stock solution, and then adjusted the pH to 6.0. These solutions were then mixed with the *L. edodes* antidote (<100 μm; 1 g/L). These mixtures were incubated at 37 °C (140 rpm). Samples were taken after 0.5, 1, 2, 3, 5, 8, 12, 16, 20, 30, 60, and 120 min incubation, and were filtered. The supernatant were analyzed to determine the remaining cadmium concentration. All experiments were done in triplicate.

### 2.6. Simulated Intestinal Fluids Treatment

The simulated intestinal fluids were prepared according to the USP guide by mixing in order the following components: monobasic potassium phosphate (6.8 g) dissolved in water (250 mL), 0.2M sodium hydroxide (77 mL), water (500 mL), and pancreatin (10.0 g, obtained from porcine pancreas). The mixture was adjusted to pH 6.8 ± 0.1 with either 0.2M sodium hydroxide or 0.2M hydrochloric acid and then diluted with water to the final volume of 1000mL. The simulated intestinal fluids were divided into a series of 100 mL solution. The cadmium ion was added to the 100 mL simulated intestinal fluids at 1, 5, 100, 200 mg/L respectively. The *L. edodes* powder (<100 μm; 1 g/L) were then added to the cadmium containing fluids, and incubated for 2h (140 rpm, 37 °C). After centrifugation, the supernatant was analyzed for the remaining cadmium.

### 2.7. Analysis

The cadmium concentration was determined using a graphitefurnaceatomicabsorption spectrometry (GFAAS, AA6300, Shimadzu, Kyoto, Japan). The cadmium adsorbed on antidote was calculated from the initial and the remaining cadmium concentration after adsorption. The surface morphology of the antidote powder was examined by scanning electronmicroscope (SEM, JSM-5610LV, JEOL, Kyoto, Japan) with 5000× magnification and an accelerating voltage of 25 keV. Elemental compositions before and after adsorption were determined by the energy dispersive spectrometry (EDS, JEM, Kyoto, Japan) with 200 KV accelerating voltage. The chemical property of the antidote surface was characterized using Fourier transform infrared (FTIR) analysis (Nicolet 5700 Thermo, MA, USA). A mass ratio of the sample to KBr at 1:100 was used for the preparation of the disks, and the spectral range of 4000 cm^−1 ^ to 400 cm^−1^ was used for FTIR analysis.

## 3. Results and Discussion

### 3.1. Weak Acidic pH Favored Cadmium Adsorption/Removal

**Figure 1:**
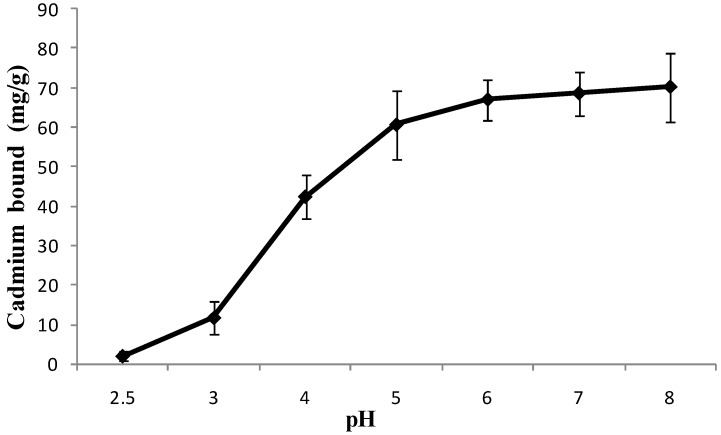
Effect of pH on cadmium binding. Conditions: Initial cadmium, 100 mg/L; antidote dose, 1 g/L; temperature, 37 °C; incubation time, 2 h.

Notes: The statistical significance between cadmium bound and pH was remarkable difference (*p* < 0.05).

The pH level may affect the functional groups (hydroxyl, carboxylate and amino groups) of the antidote, the stability of metal complex [1,14], and eventually the effectiveness of the antidote for detoxification of heavy metal poison. Indeed, the antidote will encounter a wide range of pH environment when administered to humans, as the human gastrointestinal pH varies from strong acid to weak base (pH 2–8) [11,15,16]. To this end, the pH effect was evaluated for cadmium adsorption by *L. edodes* powder (Figure 1).

Little if any cadmium adsorptions were observed when the pH was less than 2.5; the cadmium adsorption increased from 0 to 65.12 mg/g when the pH increased from 2.5 to 6.0; while no further increase of adsorption (reached to plateau) when the pH further increased from 6.0 to 8.0. These results suggested that weak acid conditions favor effective cadmium adsorption by *L. edodes*.

This little or no cadmium adsorption under pH 2.5 may be attributed to the effective competition of hydrogen ions (proton) for the available binding sites of *L. edodes* powder. When the pH increased from 2.5 to 6.0, however, the weak acid conditions provided favorable conditions for the ionization of cadmium (Cd^2+^) that could readily bind with the negative charged binding sites of *L. edodes* antidote [17]. Due to its low pH (<2.5) and a relatively short food retention, the stomach seems to not be a significant organ for cadmium adsorption by *L. edodes*. On the contrary, the small intestine has a weakly acidic environment and a long food retention period, providing a primary organ for detoxification of cadmium by *L. dodes* antidote.

### 3.2. Smaller Antidote Powder Size Improved Cadmium Removal 

**Figure 2:**
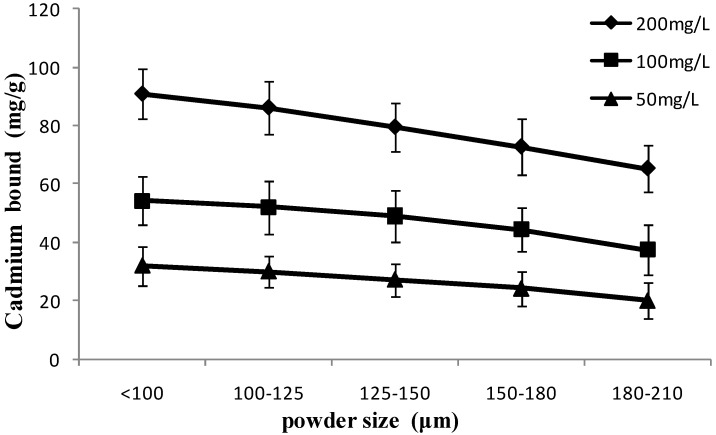
Effect of antidote powder size on cadmium adsorption. Conditions: 50, 100 and 200 mg/L; pH, 6; antidote dose, 1 g/L; temperature, 37 °C; incubation time, 2 h.

Notes: The statistical significance between cadmium bound and powder size in all three concentrations was significant (*p* < 0.01).

The surface contact of antidote plays an important role for bioadsorption. The effect of different antidote powder size (<100, 100–125, 125–150, 150–180, 180–210 µm) on cadmium adsorption/removal was investigated using three different initial cadmium concentrations (Figure 2).

At 100 mg/L initial cadmium, the amount of cadmium bound to the antidote decreased from 54.28 mg/g to 37.45 mg/g as the powder size increase from less than 100 µm to 180–210 µm. The adsorption data in Figure 2 showed that cadmium adsorbed/removed was approximately negative linear relation with the antidote powder size. It is believed that smaller powder size might enhance specific surface area, favoring the effective interaction between the antidote particles and cadmium ions [18]. Additionally, a similar relationship was observed between the antidote powder size, the adsorption/removal of cadmium, and the binding capacity per unit of antidote when the initial cadmium concentration was 50 and/or 200 mg/L.

### 3.3. Initial Cadmium Concentration Affected Effective Adsorption/Removal

**Figure 3:**
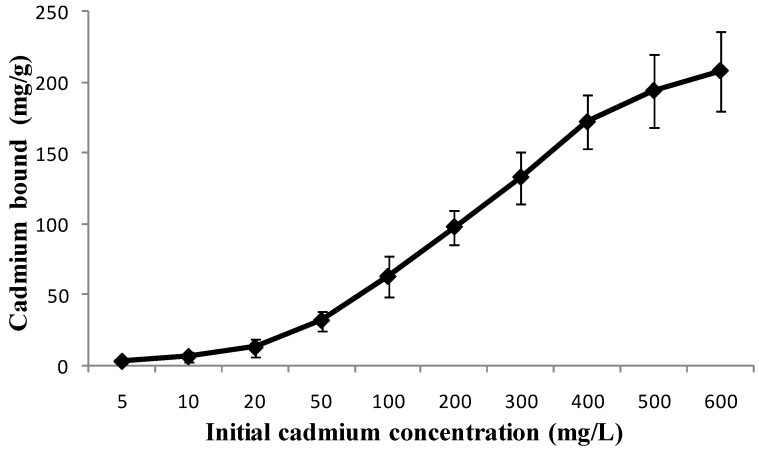
Effect of the initial cadmium concentration on adsorption. Conditions: pH, 6; antidote dose, 1 g/L; temperature, 37 °C; incubation time, 2 h.

Notes: The statistical significance between cadmium bound and initial cadmium concentration was significant (*p* < 0.01).

A series of solutions with different initial cadmium concentrations (5 to 600 mg/L) were used to evaluate the effect of initial cadmium concentration on effective adsorption/removal by *L. edodes* (Figure 3). At the initial cadmium concentration of 5–20 mg/L, a low specific binding (less than 10 mg/g) of *L. edodes* was observed. However, when the initial cadmium concentration was in the range of 20–400 mg/L, the specific cadmium adsorption of *L. edodes* increased rapidly, forming an approximately linear curve. To the higher end of initial cadmium concentration of 400–600 mg/L, the uptake speed decreased gradually. This phenomenon could be that an increase in the amount of cadmium ions caused competition for the available binding sites on the surface of antidote [16,19]. At higher concentration, the cadmium ion density was enhanced on the antidote surface, which promoted the integration between cadmium and the binding sites. In other words, high cadmium concentration plays a key role in overcoming of the mass transfer resistance between the aqueous and solid phases [15,20].

### 3.4. Cadmium Ion Binds to L. edodes Rapidly

**Figure 4:**
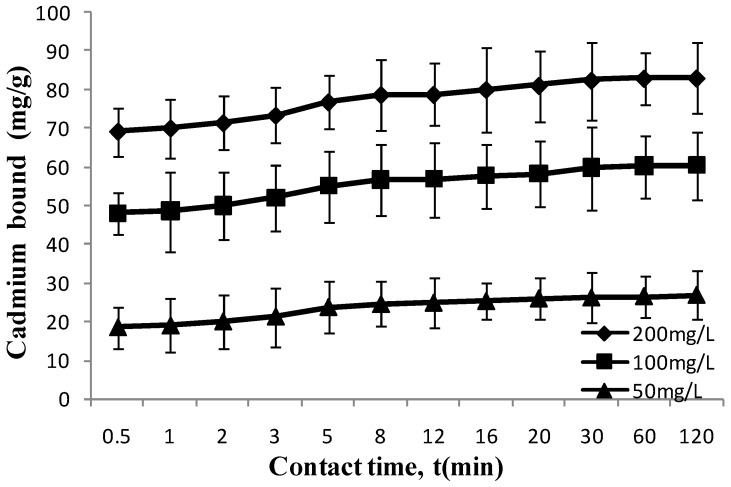
Effect of contact time on cadmium adsorption. Conditions: initial cadmium concentrations: 50, 100 and 200 mg/L for; pH, 6; antidote dose, 1 g/L; temperature, 37 °C; incubation time, 2 h.

Notes: The statistical significance between cadmium bound and contact time at all three concentrations was remarkable (*p* < 0.05).

The adsorption rate is an important parameter for an antidote to detoxify cadmium. To evaluate the potential of *L. edodes* to serve as an antidote for cadmium intoxication, three different initial cadmium concentrations (50, 100, 200 mg/L) were used to study the contact time requirement of *L. edodes* for efficient cadmium removal (Figure 4). The cadmium adsorption by *L. edodes* took place rapidly. Specifically, 19.77, 48.33 and 69.72 mg cadmium was adsorbed by *L. edodes* in just one minute for the solutions containing 50, 100, and 200 mg/L initial cadmium, respectively. In fact, the first minute’s adsorption accounted for 84% of the total adsorbed cadmium. Further increase contact time had an inconspicuous effect on cadmium adsorption. The slow adsorption after one minute may be attributed to the transfer resistance of cadmium ion to the antidote particles [21]. Nevertheless, this rapid adsorption behavior of *L. edodes* is a positive indication for its usefulness in the treatment of acute cadmium intoxication.

### 3.5. Hydroxyl, Carboxyl and –NH Groups of Polysaccharides and Proteins Provide the Sites for Cadmium Binding

**Figure 5:**
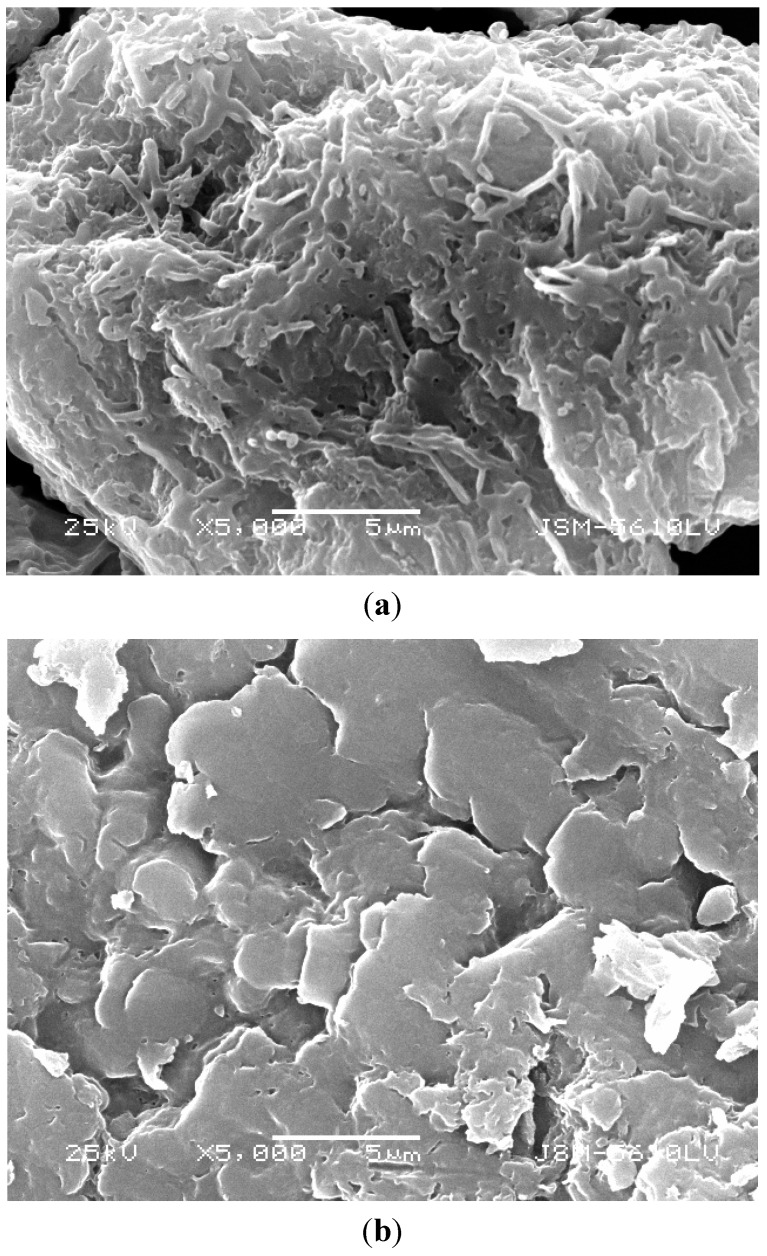
Surface morphology of *L. edodes*. (**a**) before cadmium adsorption; (**b**) after cadmium adsorption.

As shown in Figure 5, there was a significant change of the fruiting body surface of *L. edodes* when bound to cadmium ions. Before binding (Figure 5a), the surface was rough and sponge-like. These surface properties could provide large specific surface area, reduce the diffusion resistance and facilitate mass transfer, contributing to the adsorption process. The surface of the cadmium-loaded *L. edodes* (Figure 5b) was smooth. Chemical interactions between the functional groups of the *L. edodes* and cadmium ions might be responsible for the observed surface structure changes.

**Figure 6:**
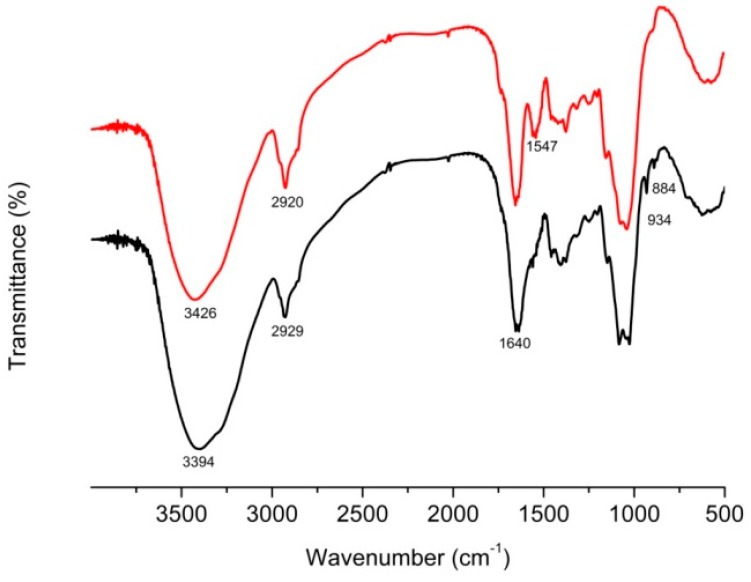
Fourier transform infrared (FTIR) spectrum of *L. edodes*. Bottom black line, *L. edodes* surface; Top red line, Cd-loaded *L. edodes* surface.

To understand the chemical interactions of *L. edodes* and cadmium ion, a Fourier transform infrared analysis was used to obtain functional group information about the *L. edodes* surface, with the assumption that the energy absorption bands should be different before and after cadmium adsorption [12]. The primary components of *L. edodes* are polysaccharides (pectin, cellulose, hemicellulose and chitin), which comprise a large amount of hydroxyl groups that can be reflected by the strong and wide absorption peak at 3400 cm^−1^. As demonstrated in Figure 6, the hydroxyl absorption peak around 3394 cm^−1^ shifted to 3426 cm^−1^ after cadmium absorption, indicating that the hydroxyl groups had been changed from multimer to monopolymer or even a dissociative state [22]. In addition, the characteristic absorption peak at 1638 cm^−1^ indicated the presence of the carboxyl groups of the pectin component. The new absorption peak appeared at 955–915 cm^−1^ after cadmium absorption might be due to the structural changes caused by carboxyl vibrations [23]. Furthermore, a new peak observed at about 1547 cm^−1^ indicated that there was an amide group–NH stretching, which suggested that proteins of *L. edodes* were also involved in the cadmium bonding reaction [24]. These results indicated that the polysaccharides and proteins present on the surface of *L. edodes* provide the opportunity to bind cadmium ions through chemical interactions.

**Figure 7:**
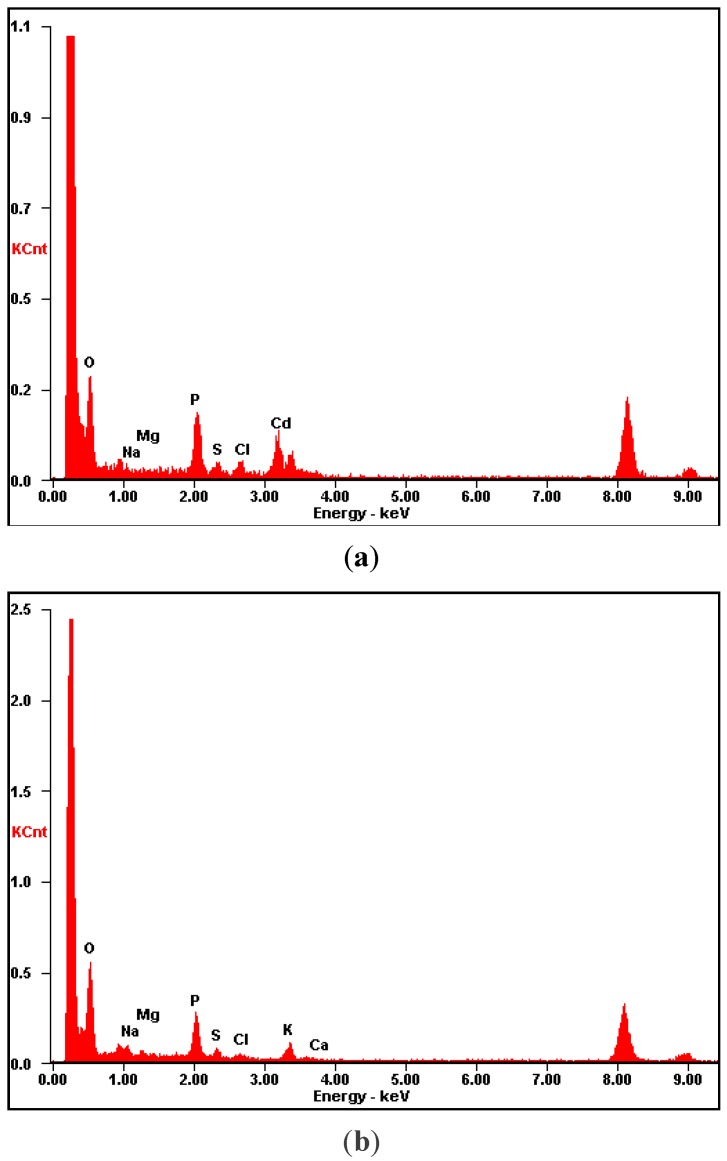
EDS spectrums of *L. edodes*. (**a**) before cadmium adsorption; (**b**) after cadmium adsorption.

The energy dispersive spectrometries of *L. edodes* powder before and after adsorption are shown in Figure 7, which indicated that carbon and oxygen are the predominant elements of the antidote. It is clearly seen that potassium and calcium peaks were not detected and a new cadmium peak appeared after biosorption. Thus, there was a possible cation exchange in the cadmium biosorption process, which had been reported by Davis [25].

### 3.6. Cadmium Adsorption Fitted well with the Languir and Freundlich Models

In order to predict the adsorption behavior of the antidote, it is necessary to establish an appropriate mathematical model to optimize the adsorption process for efficient cadmium removal. Toward this goal, the following commonly used Langmuir and Freundlich models [26] were used to evaluate cadmium adsorption process:

$$\frac{C_{e}}{q_{e}}=\frac{1}{q_{\text{max}}K_{L}}+\frac{1}{q_{\text{max}}}C_{e}\;\text{lg}\;q_{e}=\text{lg}K_{F}+\frac{1}{n}\text{lg}\;C_{e}$$

where *C_e_* is the equilibrium concentration of cadmium ion (mg/L); *q_e_* is the amount of cadmium ion adsorbed at equilibrium (mg/g); *q_max_* is the maximum amount of cadmium ion per unit mass of adsorbent (mg/g); *K_L_* (L/mg) is the Langmuir absorptionconstant representing energy of absorption; *K_F_* and n are the Freundlich equilibrium constants indicative of adsorption capacity (mg/g) and adsorption intensity (g/L).

The related parameters of Langmuir and Freundlich model for cadmium adsorption were shown Table 1. The cadmium adsorption fitted well with both models, with their simulated linear correlation coefficients were greater than 0.97. Moreover, the correlation coefficient of Freundlich model fitted better than that of Langmuir model. The maximum uptake q_max_ of cadmium was 333.33 mg/g at pH 6. The antidote q_max_ value was higher than that reported for cadmium binding *in vitro* by therapeutic drugs such as penicillamine, activated carbon, or sodium thiosulfate, which display absorption capacities of 3.2, 4.0, 328.8 mg/g, respectively. In addition, the adsorption intensity n calculated by the Freundlich equation was 1.308, which lies in the 1–10 range, suggesting a favorable binding by *L. edodes* [27]. Therefore, the relatively low-cost and high cadmium adsorption affinity of *L. edodes* make it an attractive potential antidote for the removal of cadmium from theintestine.

**Table 1:** Biosorption equilibrium parameters obtained from Langmuir and Freundlich isotherms.

Model Parameters	Langmuir	Model Parameters	Freundlich
q_max_(mg/g)	333.33	n (g/L)	1.308
K_L_(L/mg)	0.002	K_F_(mg/g)	2.72
R^2^	0.978	R^2^	0.981

Notes: q_max_: maximum amount of cadmium ion per unit mass of adsorbent; K_L_: absorption energy; R^2^: linear correlation coefficients; n: adsorption intensity; K_F_: adsorption capacity.

### 3.7. Effective Detoxication of Simulated “Cadmium Contaminated” Intestinal Fluids

The effect of *L. edodes* (1 mg/L) on cadmium removal from the simulated intestinal fluids was evaluated using four different initial cadmium concentrations (Table 2). After treatment with the antidote powder, the cadmium ion concentrations in the simulated intestinal fluids decreased by at least 50%. Specifically, 50.8%, 61.73%, 68.6% and 80% cadmium were removed from the simulated intestinal fluids with the initial cadmium concentration of 200 mg/L, 100 mg/L, 5 mg/L and 1 mg/L, respectively. These results suggested that the antidote powder was effective in cadmium detoxication, especially in low concentration.

**Table 2:** Evaluation of cadmium removal in the simulated intestinal fluids.

Before Treatment (Cadmium, mg/L)	1	5	100	200
After treatment (cadmium, mg/L)	0.2	1.57	39.27	98.33
Removal efficiency (%)	80	68.6	61.73	50.8

## 4. Conclusions

As an antidote, *L. edodes* powder effectively removed cadmium ions *in vitro* from a simulated gastrointestinal fluid with a pH of 6.8 (±0.1). A first minute removal capacity of 19.77, 48.33 and 69.72 mg was observed for initial cadmium concentrations of 50, 100, 200 mg/L, respectively. The amount of cadmium bound to the antidote, however, decreased from 54.28 mg/g to 37.45 mg/g when the powder size increased from <100 to 180–210 μm when the initial cadmium concentration was 100 mg/L. The rough and sponge-like surface of *L. edodes* observed by scanning electron microscopy was more conducive to the adsorption process. FTIR analysis showed that the functional groups from the polysaccharides and proteins of *L. edodes*, such as hydroxyl, carboxyl and –NH groups, provided the necessary sites to bind cadmium ions through chemical interactions, including cation exchange, as observed from the EDS spectra. Our results from the simulated intestinal fluids study showed that *L. edodes* was effective for cadmium detoxication, especially at low concentration. These findings are considered to be therapeutically beneficial for clinical treatment of cadmium intoxication.

## Abbreviations

*L. edodes*: *Lentinus edodes*
FTIR: Fourier transform infrared analysis
DMSA: dimercaptosuccinic acid
DMPS: dimercaptopropanesulfonate
EDTA: ethylenediaminetetraacetic acid
EDS: energy dispersive spectrometry
GFAAS: atomic absorption spectrometry
SEM: scanning electron microscope
